# Industry - from sponsor to provider?

**DOI:** 10.1080/21614083.2017.1395672

**Published:** 2017-11-01

**Authors:** Reinhard Griebenow

**Affiliations:** ^a^ European Board for Accreditation in Cardiology (EBAC) Advisory Committee, Cologne, Germany

In recent years the role of industry in the funding of medical education has been changing. While device manufacturers have announced their decision to modify their rules for funding of participants from 2018 onwards [1], the pharmaceutical industry presents itself as having moved from promotion of products to promotion of education.

This is the first time that a group of industry employees (and some are even stakeholders in their company) makes this concept public and open to discussion on a global level. It should be noted that this perspective has not been introduced by a member of the academic medical community or by any regulator.

In their paper [2] published in this issue of *JECME*, the authors describe four scenarios of continuing medical education (CME) (Categories A–D) with varying involvement of industry in planning and delivery of CME. The authors fail to point out that their Category D scenario describes the already defined setting of sponsored CME, widely accepted for accreditation by major accreditors for many years, under the condition that funding does not imply any influence of the sponsor on selection of topics, content, speakers or educational format [3,4].

Why then should we extend accreditation to educational activities with direct involvement of industry in planning and delivery of the activity (Categories A–C activities)? The authors do not provide us with a satisfactory answer.

Industry is already an important and indispensable provider in clinical research and thereby in the content of CME. Currently about 80% of all trial patients have been in clinical trials sponsored by industry [5]. However, there is published evidence that industry withholds a substantial amount of clinical study data from public scrutiny [6] and/or tries to influence its publication [7]. Thus, the medical community would probably unanimously welcome industry as a partner in ethically conducted medical research, but there is still much to do to improve transparency in these areas. In the case of CME, where evidence is put into perspective in clinical decision-making, there is a current consensus that this should fall exclusively under the responsibility of physicians and should not be influenced in any way by institutions or processes that are dominated by commercial interests.

This has nothing to do with “managing one bias by introducing another” or “building silos” or blaming industry, but just describes the response to the inevitable behaviour of industry that is a consequence of the economic framework set by governments in most developed countries.

Even more importantly, this separation of roles [3,4] constitutes the only way of maintaining the credibility of the medical profession in its patient-physician relations in the pursuit of better health outcomes.

The degree of influence that industry has on research can never be accepted in CME and the analogy between research and CME, as put forward by the authors, is false because of another fundamental difference. While the common ground for physicians and industry in conducting clinical trials is determined by a clearly defined, internationally agreed and accepted methodology, this is completely lacking in the delivery of CME, where we do not even have consensus on how to translate evidence into language.

The authors nevertheless conclude that the European Accreditation Council for CME (EACCME) should change its approach and consider CME with direct involvement of industry for accreditation (Categories A to C). It is unclear why they have targeted just one European international accreditor to be the first to breach the fundamental principles and rules of CME accreditation.

Since the EACCME has no monopoly of provision of accreditation or overriding legal legitimacy in accreditation, it is not in a position to impose recognition of its accreditation decisions on other European national regulators. Any such dramatic change in its relationship with the pharmaceutical industry could fundamentally undermine its status and lead to the rejection of recognition of any EACCME accreditation decision by the relevant European national authorities as well as major overseas accreditors.

In conclusion, this article provides no evidence for the need to change the approach of regulators to industry-provided education in their Categories A–C and hence as regards accreditable CME, it is “no time for change”.

## Supplementary Material

Declaration_of_coi-_Prof_Griebenow.pdf
