# Supplementary material for: Industry - from sponsor to provider?
Source: J Eur CME. 2017 Nov 1;6(1):1395672. doi: 10.1080/21614083.2017.1395672 (PMC5843077; doi:10.1080/21614083.2017.1395672)
Supplement: Declaration_of_coi-_Prof_Griebenow.pdf [file ZJEC_A_1395672_SM9828.pdf]

Prof. Reinhard Griebenow, MD, PhD

Declaration of interests

1. **Financial: 0**

2. **Nonfinancial:**
   Praxis Rheingalerie, Cologne
   Former Assistant Medical Director and Head of Training, Department of Cardiology, Angiology and Diabetology, Municipal Hospital Cologne (Merheim), University of Cologne, Germany
   Chairman of the Board, ECSF
   Chairman, EBAC Advisory Committee
   Vice-chair, Continuing Medical Education-European Accreditors (CME-EA)
   Head, Academy for Training and Education, Chamber of Physicians North-Rhine
   Member of the Board, Chamber of Physicians North-Rhine
   Member, Standing Committee for CME, German Medical Association
   Member, Education Committee, German Cardiac Society
   Member, Editorial Board, JECME
   Member, Committee for Training and Education Politics, Marburger Bund
   Member, AMEE CPD Committee
